# A database of 5305 healthy Korean individuals reveals genetic and clinical implications for an East Asian population

**DOI:** 10.1038/s12276-022-00871-4

**Published:** 2022-11-02

**Authors:** Jeongeun Lee, Jean Lee, Sungwon Jeon, Jeongha Lee, Insu Jang, Jin Ok Yang, Soojin Park, Byungwook Lee, Jinwook Choi, Byung-Ok Choi, Heon Yung Gee, Jaeseong Oh, In-Jin Jang, Sanghyuk Lee, Daehyun Baek, Youngil Koh, Sung-Soo Yoon, Young-Joon Kim, Jong-Hee Chae, Woong-Yang Park, Jong Hwa Bhak, Murim Choi

**Affiliations:** 1 Interdisciplinary Program in Bioengineering, Graduate School, Seoul National University, Seoul, 03080 Republic of Korea; 2 Department of Biomedical Sciences, Seoul National University College of Medicine, Seoul, 03080 Republic of Korea; 3 Department of Biomedical Engineering, College of Information and Biotechnology, Ulsan National Institute of Science and Technology (UNIST), Ulsan, 44919 Republic of Korea; 4 Korea BioInformation Center (KOBIC), Korea Research Institute of Bioscience and Biotechnology (KRIBB), Daejeon, 34141 Republic of Korea; 5 Department of Bio and Brain Engineering, Korea Advanced Institute of Science and Technology (KAIST), Daejeon, 34141 Republic of Korea; 6 Department of Pediatrics, Seoul National University College of Medicine, Seoul, 03080 Republic of Korea; 7 Department of Biomedical Engineering, Seoul National University College of Medicine, Seoul, 03080 Republic of Korea; 8 Department of Neurology, Samsung Medical Center, Sungkyunkwan University School of Medicine, Seoul, 06351 Republic of Korea; 9 Department of Pharmacology, Brain Korea 21 PLUS Project for Medical Sciences, Yonsei University College of Medicine, Seoul, 03722 Republic of Korea; 10 Department of Clinical Pharmacology and Therapeutics, Seoul National University College of Medicine and Hospital, Seoul, 03080 Republic of Korea; 11 Department of Bio-Information Science, Ewha Womans University, Seoul, 03760 Republic of Korea; 12 School of Biological Sciences, Seoul National University, Seoul, 08826 Republic of Korea; 13 Department of Internal Medicine, Seoul National University Hospital, Seoul, 03080 Republic of Korea; 14 Department of Biochemistry, College of Life Science and Biotechnology, Yonsei University, Seoul, 03722 Republic of Korea; 15 Department of Genomic Medicine, Seoul National University Hospital, Seoul, 03080 Republic of Korea; 16 Samsung Genome Institute, Samsung Medical Center, Seoul, 06351 Republic of Korea

**Keywords:** Genomics, Genetic databases

## Abstract

Despite substantial advances in disease genetics, studies to date have largely focused on individuals of European descent. This limits further discoveries of novel functional genetic variants in other ethnic groups. To alleviate the paucity of East Asian population genome resources, we established the Korean Variant Archive 2 (KOVA 2), which is composed of 1896 whole-genome sequences and 3409 whole-exome sequences from healthy individuals of Korean ethnicity. This is the largest genome database from the ethnic Korean population to date, surpassing the 1909 Korean individuals deposited in gnomAD. The variants in KOVA 2 displayed all the known genetic features of those from previous genome databases, and we compiled data from Korean-specific runs of homozygosity, positively selected intervals, and structural variants. In doing so, we found loci, such as the loci of *ADH1A/1B* and *UHRF1BP1*, that are strongly selected in the Korean population relative to other East Asian populations. Our analysis of allele ages revealed a correlation between variant functionality and evolutionary age. The data can be browsed and downloaded from a public website (https://www.kobic.re.kr/kova/). We anticipate that KOVA 2 will serve as a valuable resource for genetic studies involving East Asian populations.

## Population genetics: largest genome database for Korean ethnicity to date

A database comprising whole genome and protein-coding sequences from 5305 healthy individuals of Korean ethnicity will provide an invaluable resource for genetic studies on East Asian populations. To date, most studies of disease genetics have focused on people of European descent. Now, Jong Hwa Bhak at Ulsan National Institute of Science and Technology, Murim Choi at Seoul National University, and co-workers across South Korea have compiled the Korean Variant Archive 2 (KOVA 2; https://www.kobic.re.kr/kova/), the largest genome database ever produced for the ethnic Korean population. Initial analysis of KOVA 2 confirmed genetic signals that overlap with neighboring populations in China and Japan, as anticipated from knowledge of population mixing and similar environmental influences. The team also identified two genetic variants that are strongly selected in Koreans when compared with other East Asian populations.

## Introduction

Korean individuals are known to have migrated to the Korean peninsula at least 40,000 years ago; this migration probably occurred through two routes, from northeast and southeast Asia^1,2^. Complex but constant admixture with the neighboring Chinese and Japanese populations ensued throughout history^3^, yet many studies have suggested that ethnic Korean individuals are genetically distinct from Chinese and Japanese individuals. At ~83 million people, the ethnic Korean population is the 15th largest ethnic group in the world. Especially in South Korea, modern nationwide healthcare systems can provide an opportunity to study the genetics of various diseases in this population if the appropriate genetic infrastructure is provided.

As the field of human genetics advances, more attention is being paid to non-European populations as a new venue for obtaining novel insights into the genetics and physiology of human development, physiology, and disease. Although East Asian individuals make up nearly a fifth of the world population, they comprise only 8.2% of participants in genome-wide association studies (GWAS)^4^. Likewise, control databases—compilations of apparently healthy genomes—for East Asian populations are scarce compared to those for Europeans. For example, among the 141,156 participants in gnomAD version 2, only 9977 East Asian individuals were listed, of which only 1909 were Korean individuals. Notably, small databases of genetic information from Korean individuals (with sizes of ~1000 individuals) have been released^5–7^, including one established by our group (Korean Variant Archive [KOVA]^5^). However, as the cohort sizes of human genetics studies increase, it becomes necessary to construct larger Korean control databases.

Here, we introduce KOVA 2, a Korean control database that includes 5305 individuals. Using the variant set from KOVA 2, we determined Korean-specific runs of homozygosity (ROH) regions, intervals of positive selection, structural variants, and allelic ages. As a public resource, KOVA 2 will serve as an essential tool for genetic studies of Korean and East Asian populations.

## Materials and methods

### Cohorts and sample preparation

We collected the whole-exome sequencing (WES) and whole-genome sequencing (WGS) data for Korean individuals from independent research groups in Korea (Supplementary Table 1). All sequencing data were obtained from normal tissues or blood samples following standard protocols^5^. This project was performed with the approval of the Institutional Review Board of each group (Seoul National University and others), in which all donors provided written informed consent if available. All experiments were performed on deidentified samples and in accordance with relevant guidelines and regulations.

### Variant calling

We used BWA mem v0.7.17^8^ with default options to map raw reads to the GRCh38+decoy reference sequence. After marking duplicates and sorting by coordinating with MarkDuplicatesSpark, the mapping quality was recalibrated by BQSRPipelineSpark, implemented in GATK version 4.1.3.0^9^. Qualimap v2.2.1^10^ was used to generate quality control metrics for the mapped sequence data. Single nucleotide variants (SNVs) and small insertions and deletions (indels) were then called for each sample using GATK HaplotypeCaller with the option “-ERC GVCF”. To jointly genotype samples, we created a genomicsDB using GenomicsDBImport in GATK and followed the GATK best practice guideline^9^. Briefly, SNVs and indels were recalibrated by GATK’s VQSR model to select 99.7 and 99.0% of true sites, respectively, from the training set. The detailed workflow is described in Supplementary Fig. 1. Further analyses adopted a modified version of gnomAD QC steps^11^ and were mostly performed with Hail^12^, which is an open-source Python library for genome data analysis. After merging the WES and WGS data using Hail, we excluded multiallelic variants and variants that had a genotype quality (GQ) <20, read depth (DP) <10, allelic balance (AB) <0.2, or were in low complexity regions^13^ (Supplementary Fig. 2).

### Sex inference

We inferred the sex of each sample by calculating sex chromosome ploidy, which is defined as the coverage of sex chromosomes divided by the coverage of chromosome 21. To assign X and Y ploidy cutoffs, we calculated F-stat scores based on the linkage disequilibrium (LD)-pruned biallelic SNVs (MAF >0.05, call-rate >0.99, inbreeding coefficient score ≥ −0.03 and *R*^*2*^ for LD pruning <0.1) using the “annotate_sex” function of the gnomAD Hail library with the parameters “male_threshold = 0.8, female_threshold = 0.5”. An XX karyotype was defined if X chromosome ploidy ranged between [1.7, 3.4] and [1.55, 2.45] for WES and WGS, respectively. An XY karyotype was assigned when Y chromosome ploidy ranged between [0.2, 2.3] and [0.45, 1.11] and X chromosome ploidy was below 1.65 and 1.50 for WES and WGS, respectively (Supplementary Fig. 3). In subsequent analyses, only samples assigned to the XX or XY karyotype were used. In total, 92 samples were excluded because they were determined to be of ambiguous sex.

### Relatedness inference

To remove close relatives, we estimated kinship and the probability of identity-by-descent (IBD) being zero for every pair of samples based on the LD-pruned variants with a MAF ≥0.001, call-rate >0.99, HWE *P* > 1.0 × 10^−8^, inbreeding coefficient score > −0.025, and *R*^*2*^ for LD pruning <0.1. After calculating kinship using the “pc_relate” feature^14^ in Hail, we selected the maximal independent set of samples with kinship <0.1 using the ‘maximal_independent_set^15^ from Hail. For related sample pairs, we chose the one with a higher coverage depth.

### Population structure analysis

All biallelic autosomal SNVs from our dataset and the 1000 Genomes Project Phase 3 (KG)^16^ were merged and filtered; variants were retained if they had a MAF >0.001, call-rate >0.99, HWE *P* > 1.0 × 10^−8^, and inbreeding coefficient score > −0.025. We then pruned the variants to those with an LD *R*^2^ < 0.1. To perform a principal component analysis (PCA) on the Hardy–Weinberg-normalized variants, we used the “hwe_normalized_pca” function of Hail with *k* = 30. Each sample was assigned to an ancestry, determined as the ancestry with maximum probability emitted from a random forest model trained on the KG PCA result. We removed non-Korean or Korean-outlier samples iteratively until the Chinese, Japanese, Korean, and Vietnamese populations all became distinguishable based on PCs 1 and 2.

### Sample QC

The overall process is summarized in Supplementary Fig. 2. First, we excluded samples with ambiguous clinical status or having a mean coverage depth of <40X and <10X for WES and WGS, respectively. Samples with ambiguous or abnormal sex were then excluded, as were duplicated samples and closely related samples. We further removed samples with ambiguous ethnicity, followed by samples with a Het/Hom ratio >1.8 (Supplementary Figs. 2, 4, 5). Finally, after combining the WES and WGS data, we reperformed the relatedness inference procedure to remove WES samples that overlapped or were related to WGS samples.

### Variant quality control

The overall process is summarized in Supplementary Fig. 2. Variants were considered to have violated Hardy–Weinberg equilibrium (HWE) on allelic frequency (*P* < 1.0 × 10^−6^) when the allele frequency was >0.01 or the inbreeding coefficient score was <−0.03, and those variants were removed. Functional annotation was performed by the Variants Effect Predictor (VEP) version 101^17^. For each variant, we selected the most severe functional consequences using the gnomAD package of Hail. Ti/Tv and Het/Hom scores were computed using the ‘compute_sample_qc_metric’ function implemented in Hail (Supplementary Fig. 4).

### Phasing

After carrying out sample-level and variant-level quality control, WGS data were phased with SHAPEIT4 version 4.2.2^18^. After input to SHAPEIT4, we converted the VCF file to a PLINK file format with the option “--geno 0.1 --maf 0.001” to keep SNVs with missingness <10% and MAF >0.001. We used the genetic maps for reference version hg38 that are provided by SHAPEIT4^19^. We also phased our data with Beagle 5.2 (beagle.21Apr21.304.jar)^20^, for which we used the hg38 genetic map available at the Beagle website^21^ and the reference panel created by the 1000 Genome Project.

### Runs of homozygosity (ROH)

PLINK v1.90b6.12^22,23^ was used to call ROH regions from SHAPEIT-phased data with the options “--maf 0.05 --hwe 0.00005 --homozyg --homozyg-snp 50 --homozyg-kb 500 --homozyg-density 10 --homozyg-gap 10 --homozyg-window-snp 50 --homozyg-window-missing 5 --homozyg-window-het 1 --homozyg-window-threshold 0.05”. To ensure the fair comparison of ROH intervals from KOVA 2 with other populations in the KG, the regions were called from randomly selected sets of 105 samples from KOVA 2. After merging the ROH results from KOVA 2 and KG data, we calculated F_ROH_ scores, representing inbreeding levels, using the ‘Froh_inbreeding’ function of detectRuns package version 0.9.6^24^.

### Regions of positive selection

Selected variants in positive selection sweeps were captured from phased KOVA 2 and KG data using iSAFE v1.0.7^25^ software. iSAFE uses a statistic generated from population genetics signals to precisely identify the preferred variant in a large region (~5 Mbp). A variant is favored if its iSAFE score is larger than 0.1 (*P* < 1.0 × 10^−4^), and a high iSAFE score signifies that the variant is strongly positively selected. We used iSAFE with default options (--MaxRegionSize 6000000 --window 300 --MaxRank 15 --MaxFreq 0.95 --IgnoreGaps) plus the performance-improving parameter “--vcf-cont” with random outgroup (nontarget) samples comprising 10% of the data.

### Effective population size estimation

To estimate the historical effective population size, we used IBDNe software^26^ according to the recommended protocol. Briefly, after detecting IBD segments with hap-IBD.jar^27^, we refined them through the removal of any breaks and short gaps from the segments using merge-ibd-segments.17Jan20.102.jar^28^. Finally, we used ibdne.23Apr20.ae9.jar^26^ with default options to estimate the effective population size from the refined IBD segments.

### Allele ages

Genealogical Estimation of Variant Age (GEVA) version v1beta^29^ with parameters “--Ne 10000 --mut 1e-8 --maxConcordant 500 --maxDiscordant 500” was used to estimate the ages of variants from autosomal haplotype data phased by SHAPEIT4. Allele ages were computed by the joint clock model, which combines the mutation and recombination clock models. To compare allele ages as estimated by our data with those estimated from the 1000 Genomes data, we downloaded the Atlas of Variant Age from the developer’s website^30^. Chimpanzee variants called from 25 individuals were downloaded from the Great Ape Genome Project^31^.

### Imputation of array data

Imputation of variants based on KOVA 2 was performed as previously described^7^. Variants present on the Infinium Global Screening Array (GSA-24v3-0_A1) were extracted from WGS data of 197 COVID-19 patients and imputed using Impute2^32^. Panel imputation accuracy was compared using the aggregated squared Pearson correlation coefficient (*R*^*2*^) determined between the imputed genotype dosages and the true genotypes from genome data.

### Calling of structural variants (SVs)

Manta v1.6^33^ was used to call structural variants for individual WGS samples. The convertInversion.py script provided with Manta was applied to represent inversion events in the manner of gnomAD SV v2.1^34^. Slightly different SV representations across VCF files were merged using svimmer^35^. An SV was defined as known if it overlapped with any entry in the gnomAD SV v2.1 dataset.

## Results

### Characteristics of the genetic variants in the KOVA 2 dataset

To construct a Korean control database, we collected WES and WGS data generated from multiple projects that targeted Korean individuals (Supplementary Table 1). Samples originated from the normal tissues of cancer patients (40.2%), healthy parents of rare disease patients (28.4%), or healthy volunteers (31.4%) (Supplementary Table 1). Raw reads from 6654 sequencing libraries (4258 WES and 2396 WGS) were collected, processed, and filtered according to criteria from our previous experience^5^ and other studies. Exclusion criteria for samples and variants are described in the Methods, Supplementary Tables 2,3 and Supplementary Fig. 2. After filtering out 1349 samples (20.3% of initial samples), variants from the remaining 5305 samples (3409 WES and 1896 WGS) were used in subsequent analyses. A total of 40,414,379 SNVs (874,026 coding and 39,540,353 noncoding) and 2,888,275 indels (37,663 coding and 2,850,612 noncoding) were called. From WGS data only, 144,388 CNVs (65,017 deletions, 10,956 duplications, and 68,415 others) were called (Supplementary Fig. 6).

**Fig. 1:**
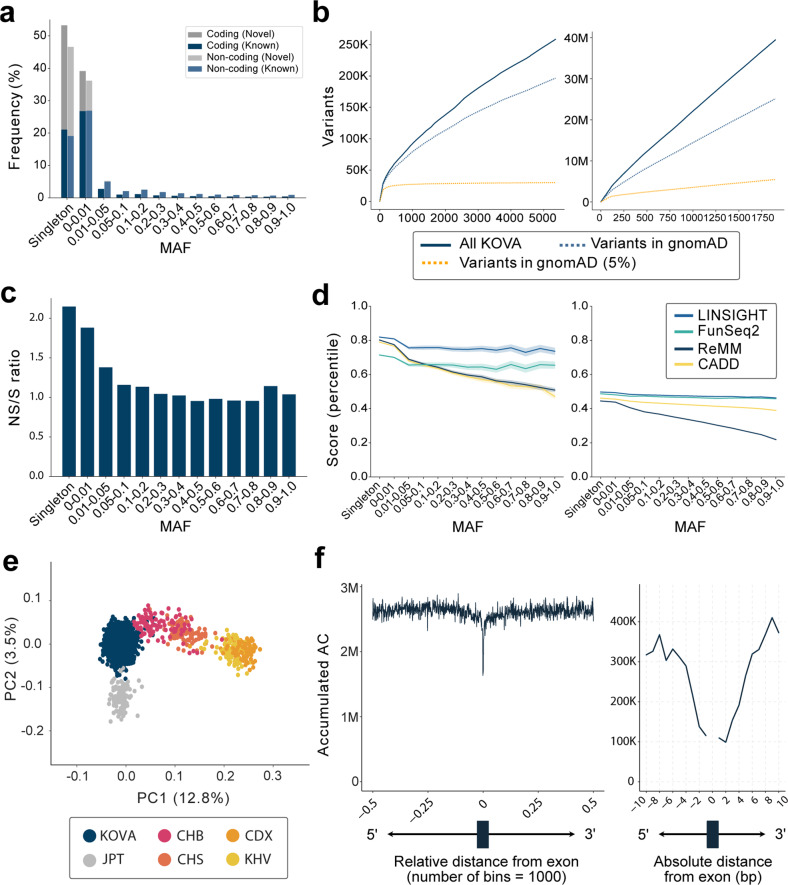
Profile of variants in the KOVA 2 dataset. **a** Variant frequency by MAF according to variant status: coding and noncoding, known and novel. **b** The number of variants identified as the number of included KOVA 2 individuals was increased and is divided by the coding (left) and noncoding (right) status. **c** The nonsilent/silent (NS/S) ratio of coding variants by MAF. **d** The patterns of variant functionality predicted by different software according to MAF are divided by the coding (left) and noncoding (right) status. As each program produces scores with different scales, and each scoring system was converted to percentiles. **e** The PCA of KOVA 2 and the neighboring East Asian populations. CHB Han Chinese individuals from Beijing, CDX Chinese individuals Dai from Xishuangbanna, JPT Japanese individuals from Tokyo, CHS Han Chinese individuals from South China, and KHV Kinh individuals from Ho Chi Minh City. **f** Intron variant burden according to the relative (left) or absolute (right) distance from exons. The X-axis bins in **c** and **d** are the same as those in **a**.

Evaluation of the minor allele frequency (MAF) distribution revealed high enrichment of rare variants (<1%) that included a larger proportion of novel variants not found in control gnomAD v3.1^11^ database (Fig. 1a, Supplementary Table 4, and Supplementary Fig. 7). As seen in other population datasets, adding data from more Korean individuals was not sufficient to saturate newly discovered variants, whether in coding or noncoding regions, whereas common coding variants (>5% frequency in gnomAD v3.1) were quickly saturated at <500 samples (Fig. 1b). Interestingly, common noncoding variants still displayed an increasing trend by analyzing 1800 WGS samples. This finding indicates that larger sample size is needed to fully cover this group of variants (Fig. 1b). As expected, variant function indicators, such as the nonsilent/silent (NS/S) ratio, CADD^36^, ReMM^37^, FunSeq2^38^, and LINSIGHT^39^, all showed increased functionality as MAF decreased (Fig. 1c, d). PCA located KOVA 2 samples in a cluster that was separate from the samples from Japanese, northern Chinese, southern Chinese, and Southeast Asian individuals (Fig. 1e and Supplementary Fig. 5). Finally, we found that the distribution of variants in the proximal intron regions indicates strong selection against any base change as variants approach exon‒intron boundaries (Fig. 1f). These results demonstrate the high quality of the KOVA 2 variant set.

### Pathogenic variants

To determine if the KOVA 2 set contains variants that have previously been annotated as pathogenic, we selected KOVA-specific rare variants (MAF <0.001) in high pLI genes and compared them against ClinVar. A total of 25 variants (seven loss-of-function (LoF) and 18 missense variants) that were identified in the KOVA 2 participants were labeled as “likely pathogenic” or “pathogenic” in relation to diseases that follow a dominant inheritance pattern (Table 1). This observation suggests that these variants may not be pathogenic, as they were previously thought to be. Alternately, since KOVA 2 is composed of three main types of individuals, i.e., healthy volunteers, normal genomes of cancer patients, and healthy parents of rare disease patients, one may argue that the variants may predispose carriers to develop cancer or their children to manifest rare diseases.

**Table 1:** Missense and high confidence (HC) loss-of-function (LoF) variants identified in KOVA 2 that are pathogenic or likely pathogenic in ClinVar but not found in gnomAD.

Variant class	Locus (hg38)	Base change	AC	AN	AF	Carrier type*	Gene symbol	pLI	ClinVar^**^	Dominant or Recessive***	ClinVar condition
LoF (HC)	chr3:128481942	CG > C	1	12,234	0.8 × 10^−4^	C	*GATA2*	0.98	P	D	Lymphedema, primary, with myelodysplasia; GATA2 deficiency with susceptibility to MDS/AML
	chr3:41236467	CAG > C	1	12,148	0.8 × 10^−4^	V	*CTNNB1*	1.00	P	D	Mental retardation, autosomal dominant 19; Inborn genetic diseases
	chr6:79026060	A > C	1	12,150	0.8 × 10^−4^	P	*PHIP*	1.00	P	D	Developmental delay, intellectual disability, obesity, and dysmorphic features
	chr7:128846444	C > T	1	12,136	0.8 × 10^−4^	C	*FLNC*	1.00	P	D	Myofibrillar myopathy, filamin C-related; Myopathy, distal, 4; Cardiomyopathy, familial hypertrophic, 26; Dilated cardiomyopathy, dominant
	chr9:95458142	G > T	1	12,120	0.8 × 10^−4^	V	*PTCH1*	1.00	P	D	Gorlin syndrome
	chr12:868379	C > T	1	12,134	0.8 × 10^−4^	C	*WNK1*	1.00	P	D/R	Hereditary sensory and autonomic neuropathy type IIA
	chrX:40064351	G > A	1	12,224	0.8 × 10^−4^	C	*BCOR*	1.00	P	D	Oculofaciocardiodental syndrome
Missense	chr1:42927147	C > T	1	12,152	0.8 × 10^−4^	C	*SLC2A1*	0.99	LP	D/R	Not provided
	chr2:108753474	A > G	1	9310	1.1 × 10^−4^	C	*RANBP2*	1.00	P	D	Encephalopathy, acute, infection-induced, 3, susceptibility to
	chr3:123296110	G > A	1	12,122	0.8 × 10^−4^	P	*ADCY5*	0.99	LP	D/R	Inborn genetic diseases
	chr3:128483925	C > T	1	12,238	0.8 × 10^−4^	P	*GATA2*	0.98	P	D	Lymphedema, primary, with myelodysplasia; GATA2 deficiency with susceptibility to MDS/AML
	chr5:128395182	C > T	1	12,160	0.8 × 10^−4^	P	*FBN2*	1.00	C	D	Congenital contractural arachnodactyly
	chr5:138570987	T > C	3	10,530	2.8 × 10^−4^	P, V	*HSPA9*	0.97	P	D/R	Even-plus syndrome
	chr6:157206668	C > T	1	12,144	0.8 × 10^−4^	V	*ARID1B*	1.00	LP	D	Coffin-Siris syndrome 1
	chr6:3154909	C > T	1	12,116	0.8 × 10^−4^	V	*TUBB2A*	0.94	P/LP	D	Cortical dysplasia, complex, with other brain malformations 5
	chr7:150952508	G > A	1	12,128	0.8 × 10^−4^	C	*KCNH2*	0.99	LP	D	Arrhythmia; Long QT syndrome 2; Congenital long QT syndrome
	chr7:5528486	G > C	1	12,126	0.8 × 10^−4^	V	*ACTB*	0.99	LP	D	Not provided
	chr9:130872896	C > T	1	12,244	0.8 × 10^−4^	C	*ABL1*	1.00	P/LP	D	Chronic myelogenous leukemia, BCR-ABL1-positive; Lymphoblastic leukemia, acute, with lymphomatous features; Leukemia, Philadelphia chromosome-positive, resistant to imatinib
	chr9:132328351	A > G	1	12,152	0.8 × 10^−4^	P	*SETX*	0.96	P	D/R	Spinocerebellar ataxia, autosomal recessive, with axonal neuropathy 2
	chr11:119089747	G > A	1	12,144	0.8 × 10^−4^	V	*HMBS*	0.95	P	D	Acute intermittent porphyria
	chr11:119092785	G > A	1	12,138	0.8 × 10^−4^	V	*HMBS*	0.95	LP	D	Not provided
	chr12:47978736	G > A	8	10,014	8.0 × 10^−4^	C, P, V	*COL2A1*	1.00	LP	D	Spondyloepiphyseal dysplasia, Namaqualand type
	chr15:48470646	C > T	1	12,140	0.8 × 10^−4^	P	*FBN1*	1.00	LP	D	Not provided
	chr16:9840706	G > A	1	12,148	0.8 × 10^−4^	V	*GRIN2A*	1.00	P/LP	D	Epilepsy, focal, with speech disorder and with or without mental retardation;
	chr18:44951948	G > A	1	12,250	0.8 × 10^−4^	C	*SETBP1*	1.00	P	D	Chronic myelogenous leukemia, BCR-ABL1 positive; Schinzel-Giedion syndrome

^*^C: normal sample of a cancer patient, P: parent of a rare disease patient, V: healthy volunteer.

**P: pathogenic, LP: likely pathogenic.

***D: dominant, R: recessive, D/R: observed in both patterns.

### Regions of homozygosity and positive selection

**Fig. 2:**
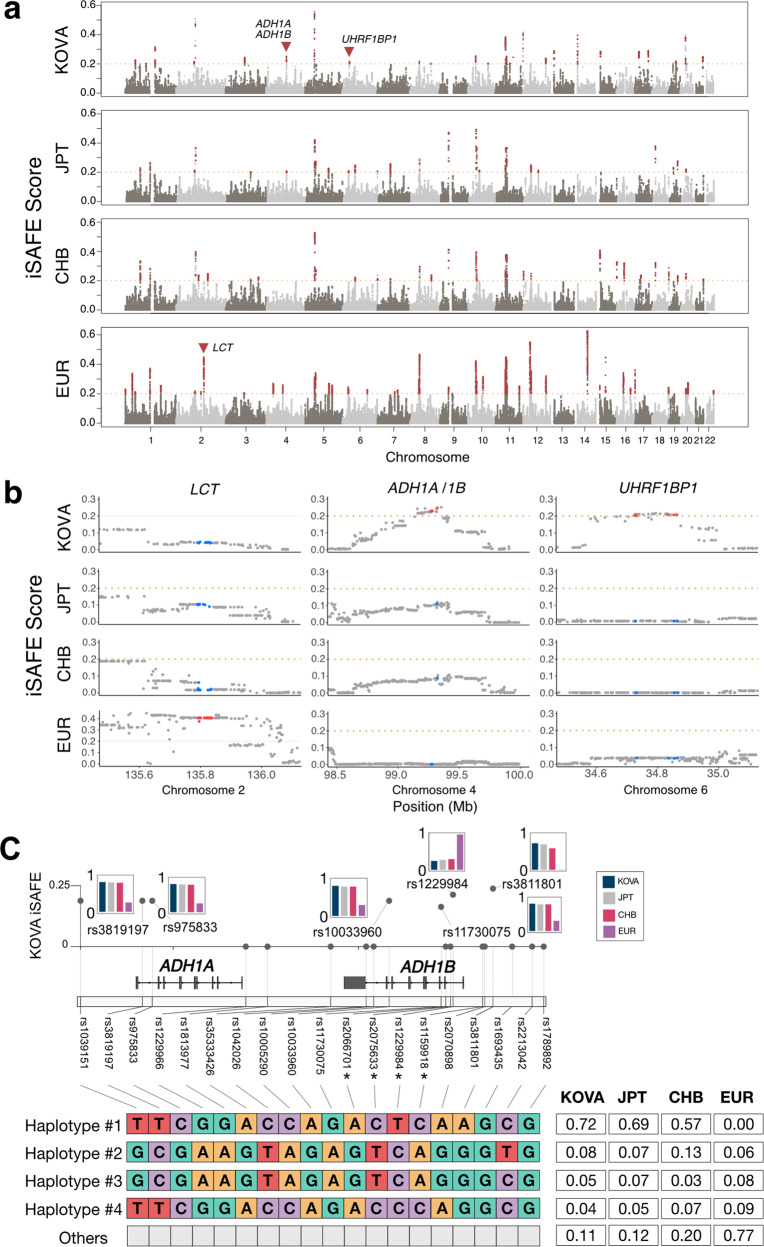
Signature of positive selection as indicated by the iSAFE score. **a** Genome-wide iSAFE values were obtained using KOVA 2, Japanese, Chinese, and European cohorts. Gene loci indicated with triangles are separately displayed in **b**. **b** Regional plots of the iSAFE values from the same set of ethnic cohorts, as marked in **a**. **c** The KOVA iSAFE scores of selected tag SNPs in the *ADH1A/1B* locus (top) and their haplotype frequencies by population (bottom). Bar plots denote the MAF of designated SNPs in each population, and SNPs with asterisks denote major markers for haplotype identification used in Han et al., 2007^42^.

Compared to populations with a higher burden of consanguinity, homozygous pathogenic variants in ROH are rarely found in an outbred population, such as the Korean population^40^. Rather, such regions can be used to signify a positive selection that was imposed on the population in the form of a selective sweep^41^. In terms of the ROH profile, the population in KOVA 2 does not deviate much from East Asian populations in general (Supplementary Fig. 8). To further characterize intervals that represent positive selection in KOVA 2, we applied the iSAFE algorithm^25^. It yielded a total of 16,272 loci that were selected in at least one population (iSAFE >0.2) and identified a number of unique loci unique to each population (172 for KOVA 2, 149 for the Japanese population, 77 for the Chinese population, and 364 for the European population) (Fig. 2a). Although the functional and expression analyses of these loci did not yield notable features, a well-known locus in *LCT* showed a strong selection signal in the European population (Fig. 2b). Interestingly, we provide evidence that two loci—*ADH1A/1B* and *UHRF1BP1*—are among the most strongly selected loci in the Korean population when compared to the Japanese, Chinese, and European populations (Fig. 2b). *ADH1A* and *ADH1B* encode alcohol dehydrogenases 1A and 1B and are known to comprise a recently selected locus in East Asian individuals^42,43^. Here, we show that the Korean population displayed the strongest signal among the East Asian populations that we evaluated. It has the highest frequency of “haplotype #1”, which represents the East Asian haplotype identified by a previous study^42^ (Fig. 2c). This signal is also reflected in the minor allele frequency of rs1229984, which was the lowest among the studied populations (Fig. 2c). The prominent Korean-specific signal we observed in *UHRF1BP1* has not been reported elsewhere, and the function of the gene remains largely unknown.

### Allele ages

**Fig. 3:**
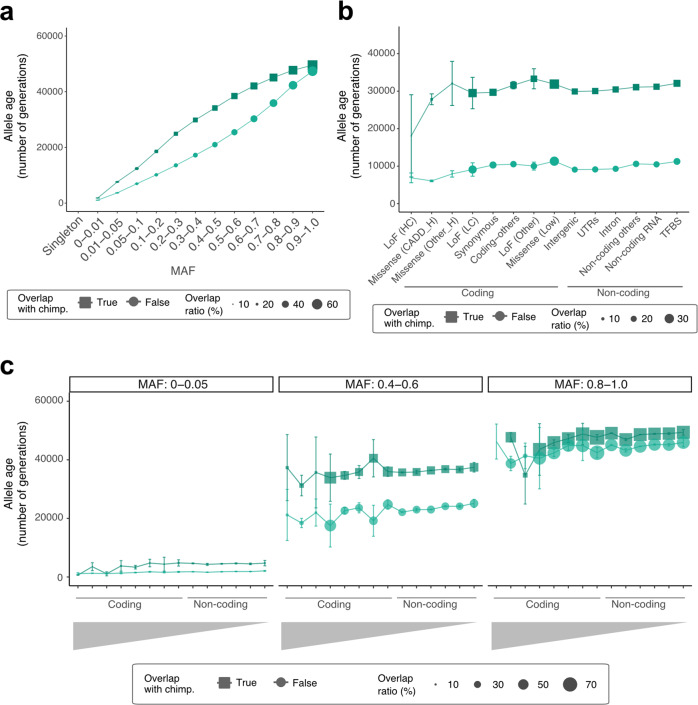
Allele ages of KOVA 2 variants. **a** Allele ages by MAF, divided by whether the allele cooccurs in chimpanzees (squares) or not (circles). **b** Allele age by predicted function, divided by whether the allele cooccurs in chimpanzees (squares) or not (circles). TFBS denotes the transcription factor-binding site. **c** Allele age by MAF and predicted function. Three MAF intervals are displayed. The X-axis bins in **c** are the same as those in **b**.

Next, we sought to determine whether KOVA 2 can be used to estimate the dates of origin for variants or allele ages and the implications of such information with regard to the function and frequency of variants. Notably, the estimation of allele ages may lead to the discovery of population-specific variants that have emerged recently. To carry out this analysis, we first phased our WGS-originated variants using a previously reported method^7,16,44^. This allowed us to estimate the population size, which came to 10–20 million. This is a value that is similar to that of the current Korean population size of ~50 million—especially given the recent population explosion (e.g., the Korean population was ~20 million in 1950 and 13 million in 1925^45^) (Supplementary Fig. 9). Next, variants with frequency >1% were used to estimate allele ages. As expected, the obtained allele ages showed a strong correlation with the MAF. The allele age was greater in variants with a high MAF or vice versa. Interestingly, variants of greater age showed higher overlap with variants from chimpanzees, suggesting that some of these variants may have a primate-level origin (Fig. 3a and Supplementary Fig. 10). Separating variants by function revealed that older allele ages and higher overlap with chimpanzees corresponded to less functionality, as indicated by annotation (Fig. 3b, Supplementary Table 5, and Supplementary Figs. 10, 11). Remarkably, high confidence LoF and high CADD score missense variants were the youngest and showed minimal overlap with chimpanzees. In addition, all functional classes of rare variants with a MAF <5% were young and did not overlap with chimpanzees (Fig. 3c and Supplementary Fig. 10). This trend was not clearly replicated when variants were categorized by pLI score (Supplementary Fig. 12). Overall, these findings suggest that most rare variants are of relatively recent origin and therefore tend to be population-specific.

## Discussion

Here, we report the establishment of the largest Korean control genome database to date, along with its genetic features and applications. KOVA 2 displayed the major features of ethnic genome databases, and we added considerable genetic information to the dataset. The variant set in KOVA 2 has been uploaded and will be shared among the community to be used as a control set in East Asian genetic studies.

First, to validate the integrity of our calling pipeline, we performed three distinct comparison evaluations. (1) A variant set from a sample that was analyzed by different sequencing techniques was compared. The variants showed a 99.4% concordance rate between HiSeq and PacBio calls and a 99.8% concordance rate between HiSeq and NovaSeq calls (Supplementary Tables 6, 7). (2) Additionally, our pipeline called 97.2% of Sanger-validated variants, and the missing calls were entirely caused by low-coverage regions (Supplementary Table 8). (3) Finally, a comparison of common variant calls across the WES and WGS platforms was performed. Among the 45,413 common coding variants (>5% frequency), 40,489 were detected by WES, and 45,118 were detected by WGS, showing 88.5% concordance (Supplementary Table 9). The missed calls were primarily from WES. This concordance is similar to one that was calculated from a recent study using 150,119 UK Biobank individuals^46^.

Within the KOVA 2 dataset, the identified variants displayed typical patterns of purifying selection and frequency-functionality relationships. The sample size does not confer enough power to cover all rare variants in the population, as is the case with other larger variant sets. However, it exhibits the best coverage of common variants in Koreans and hence better performance when imputing variants (Supplementary Fig. 13). Although KOVA 2 can serve as a control set for filtering nonpathogenic variants for rare Mendelian diseases, we identified a list of ClinVar pathogenic variants that are present at low frequency. Whether these variants are nonpathogenic in the Korean population or the genomic background enabled these carriers to evade developing the associated diseases should be further elucidated.

A combined analysis of the positive selection signatures and allele age estimation may lead to the discovery of genetic loci that have recently arisen and been selected in a population. Not surprisingly, the top signals in this Korean population overlapped with those from neighboring populations in East Asia. This finding demonstrates a recent diversion, the continuous admixture, and the similar environmental constraints that were exerted on these populations during recent evolution. Nevertheless, our results identified loci that merit further investigation. For example, we further dissected a known East Asian-selected alcohol dehydrogenase gene locus and found that it was the most strongly selected locus among the three East Asian populations analyzed here. The major haplotype in East Asian populations (“Haplotype #1” in Fig. 2c) was the most abundant in the Korean population, and among East Asian populations, the Korean population had the highest frequency of the functional variant *ADH1B* p.Arg48His (Fig. 2c). This variant is known to cause increased aldehyde production relative to its wild-type counterpart. This is due to the increased oxidation of ethanol and subsequently triggers adverse reactions, such as flushing and nausea^47^. In the long term, the variant is also protective against alcohol dependency^48^. The functional consequence of the second locus of interest, *UHRF1BP1*, remains uncertain, as it has received little study. Nevertheless, it is remarkable that associations between the variants in this gene and systematic lupus erythematosus have been repeatedly reported in East Asian populations^49–51^. This gene is most strongly expressed in the testes (Supplementary Fig. 14), making it possible to infer that it can confer selection by affecting the reproductive process in males. Looking beyond these two loci, a new algorithm based on large-scale population data may discover novel loci that were missed in our study.

Finally, we deposited the data in a genome browser and enabled the downloading of the variant set by users with a minimal registration process. The establishment of a Korean-specific variant set and comparative analysis will bolster various types of genetic and genomic studies involving East Asian populations. In addition, it will serve as a precursor for much larger genome datasets that will be available soon, especially if they can be merged with data from North Korean individuals.

## Supplementary information

Supplementary file

## Data Availability

A list of the annotated SNVs, indels, and CNVs with frequency information can be viewed and downloaded from the KOVA 2 website (https://www.kobic.re.kr/kova/).
