# Supplementary material for: A database of 5305 healthy Korean individuals reveals genetic and clinical implications for an East Asian population
Source: Exp Mol Med. 2022 Nov 2;54(11):1862–71. doi: 10.1038/s12276-022-00871-4 (PMC9628380; doi:10.1038/s12276-022-00871-4)
Supplement: Supplementary file 1 — Supplementary file [file 12276_2022_871_MOESM1_ESM.pdf]

<Supplemental materials>

A database of 5,305 healthy Korean individuals reveals genetic and clinical implications for an East Asian population

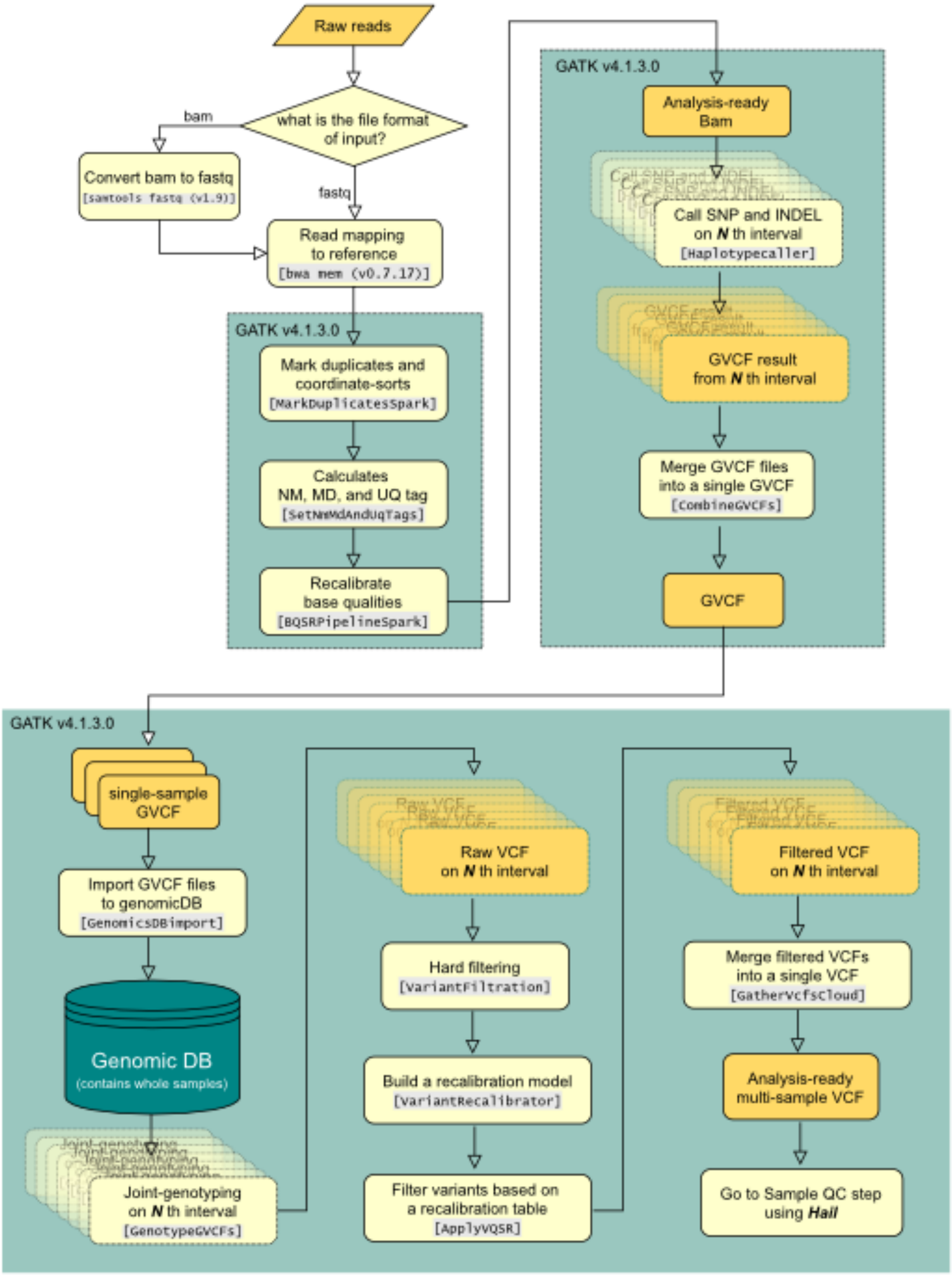

Supplementary Fig. 1. Variant calling pipeline. The name of the tool or function for each step is described in the bracket in a gray background. If the same procedure is performed in a reiterative manner, the step is depicted in overlapped boxes. The subsequent steps after BWA mapping are all proceeded using GATK v4.1.3.0.

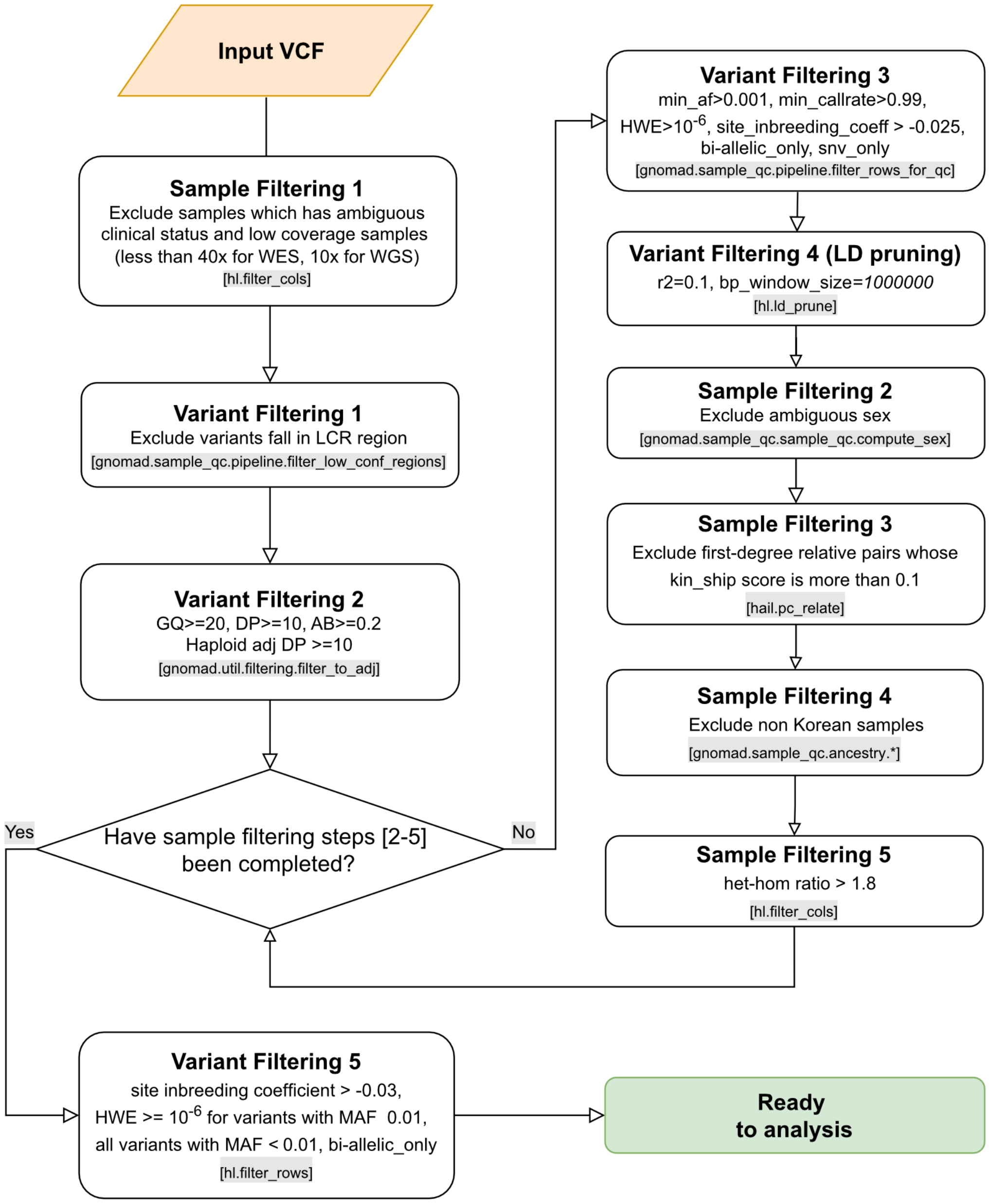

Supplementary Fig. 2. Quality control process. Input VCF is the output of the pipeline in Fig S1. Each block contains the Hail function in gray background.

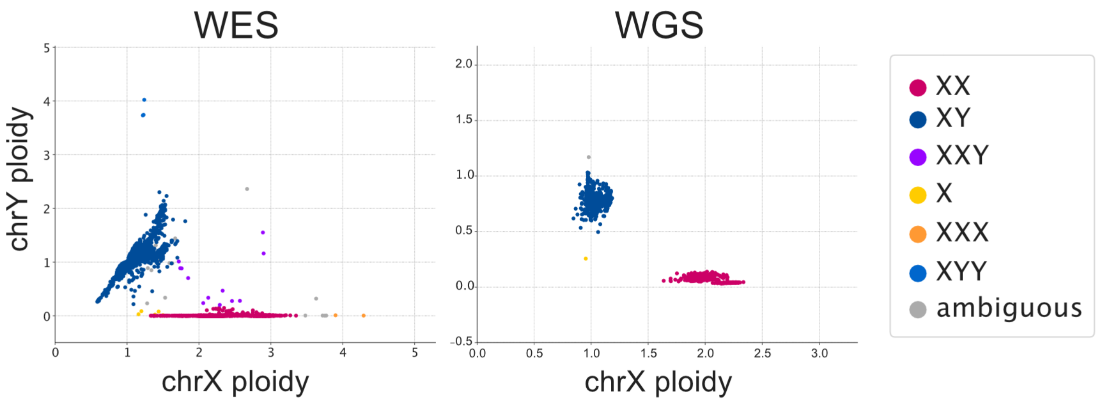

Supplementary Fig. 3. Sex inference. The X-axis and Y-axis represent the ploidy of chromosome X and Y, respectively, normalized by the coverage of chromosome 21.

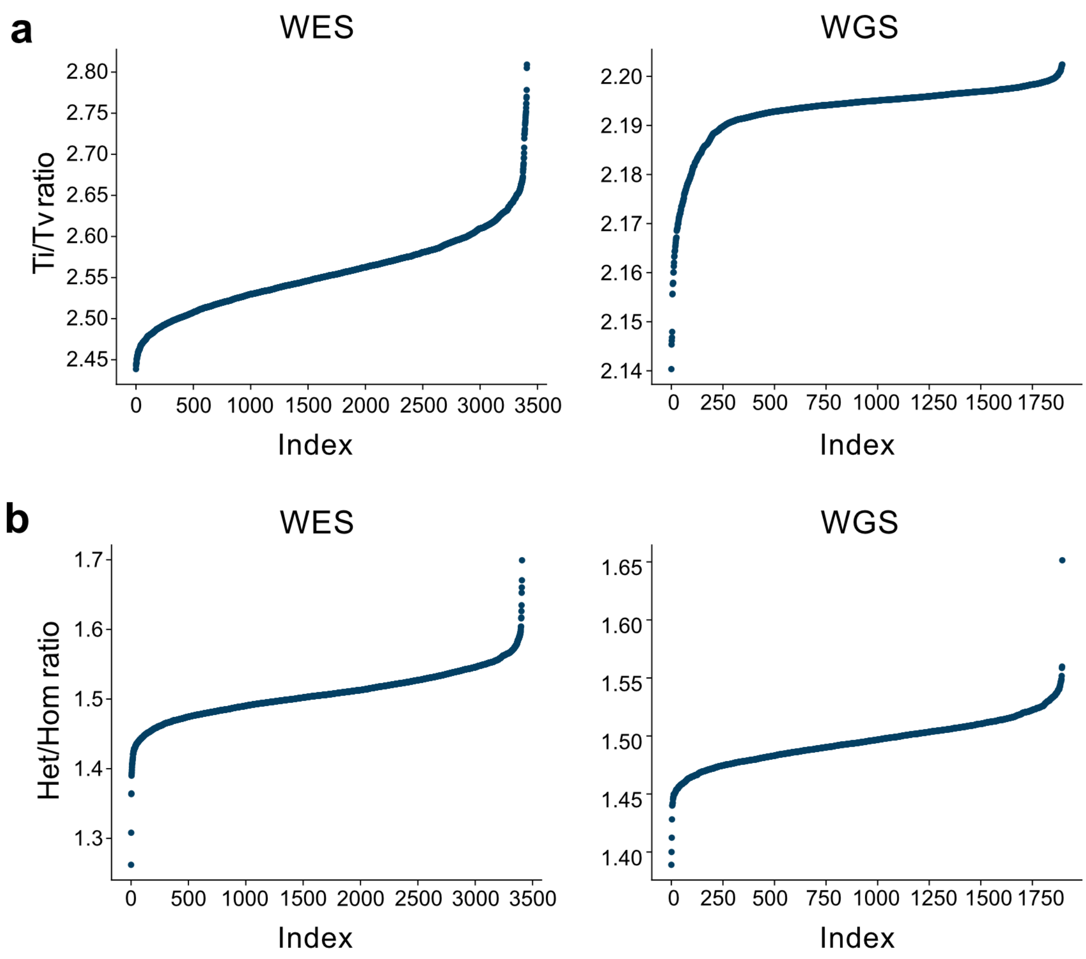

Supplementary Fig. 4. Quality control of KOVA 2 samples. (a) A transition/transversion (Ti/Tv) ratio value distribution for WES (left) and WGS (right) samples. (b) A heterozygous/homozygous ratio value distribution on WES (left) and on WGS (right) samples.

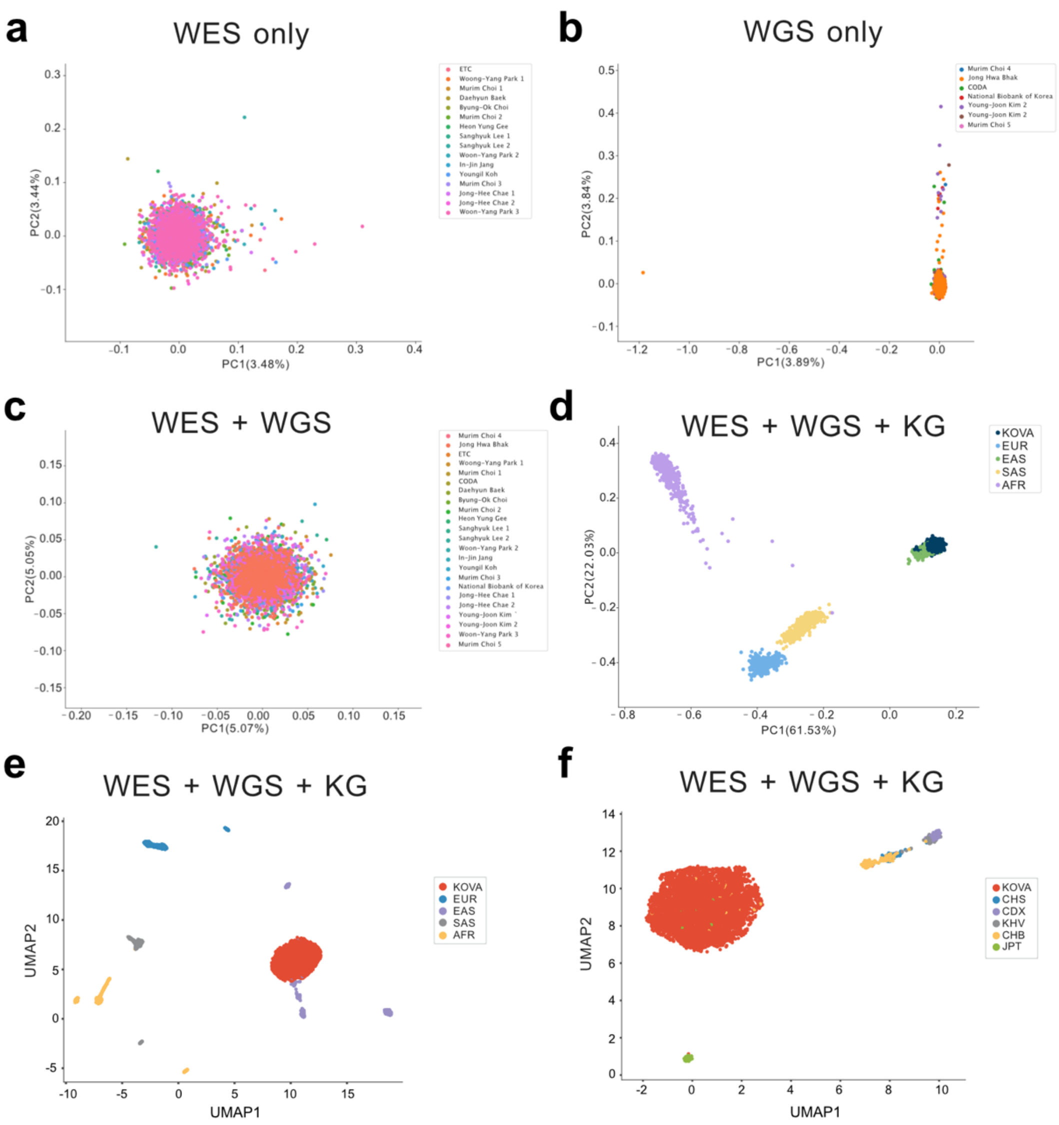

Supplementary Fig. 5. Dimension reduction analyses of KOVA 2 samples. (a) PCA of KOVA 2 WES samples. (b) PCA of KOVA 2 WGS samples. (c) PCA on WES-WGS combined samples. (d) PCA of KOVA 2 with KG individuals. (e) UMAP of KOVA 2 with KG individuals. (f) UMAP of KOVA 2 with Asian populations.

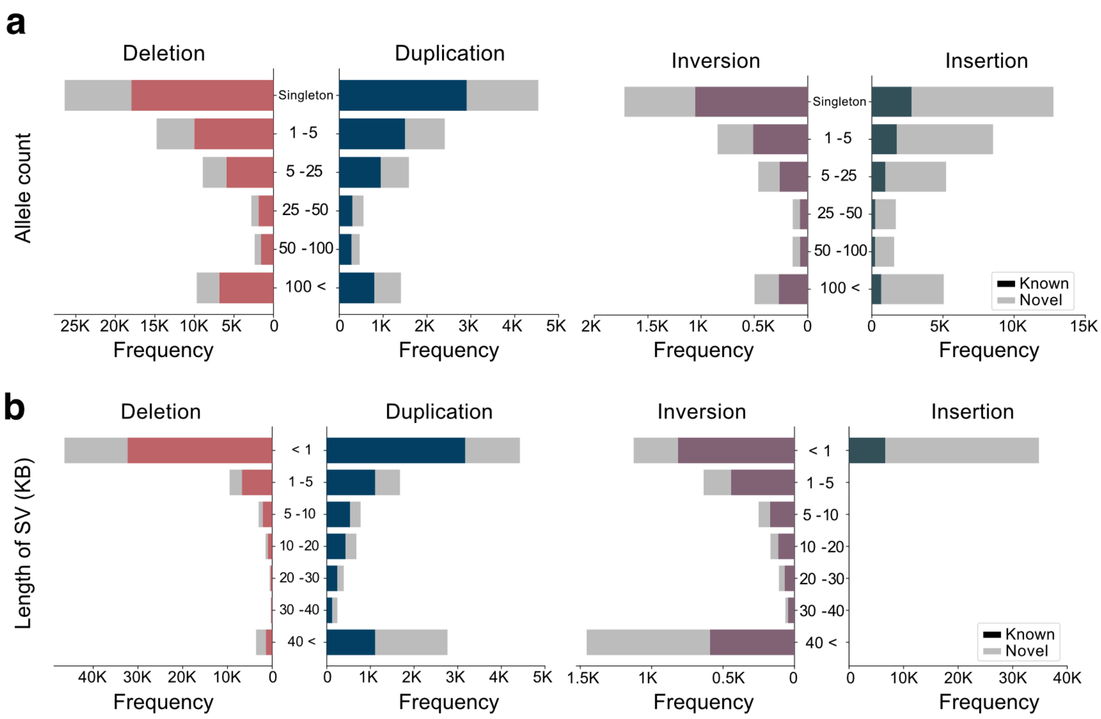

Supplementary Fig. 6. Structural variant profile of KOVA 2. (a) Number of SV variants by allele counts, divided by SV type, and known (dark-colored) and novel (gray-colored) variants according to gnomAD SV database. (b) Number of SV variants by the length of SV in kilobases.

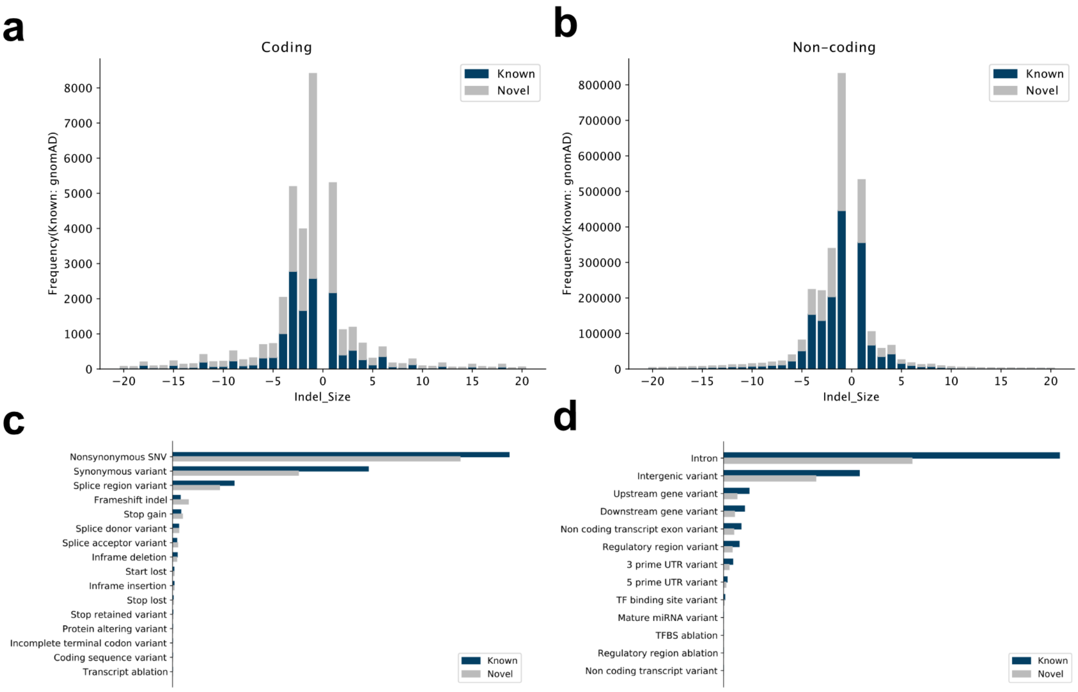

Supplementary Fig. 7. Functional annotation analysis of KOVA 2 variants. The distributions of indel sizes in (a) coding and (b) non-coding regions. The frequency of known variants is in dark blue. The number of variants by annotated function on (c) coding and (d) non-coding regions.

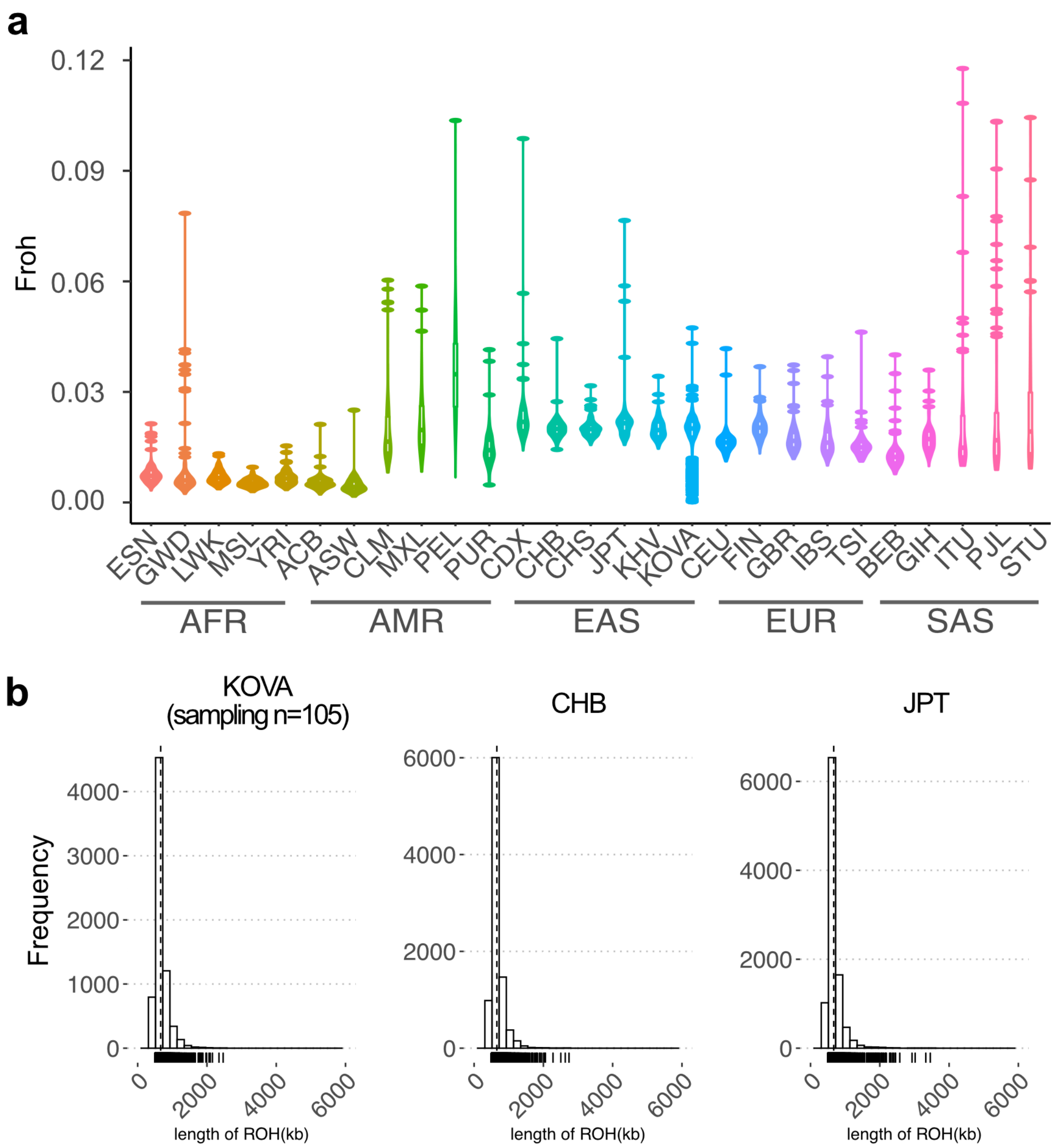

Supplementary Fig. 8. ROH profile of KOVA 2 samples. (a) Fraction of ROH per individual (Froh) by population, from KG. (b) Distribution of ROH interval length in KOVA 2, Han Chinese in Beijing (CHB), and Japanese (JPT).

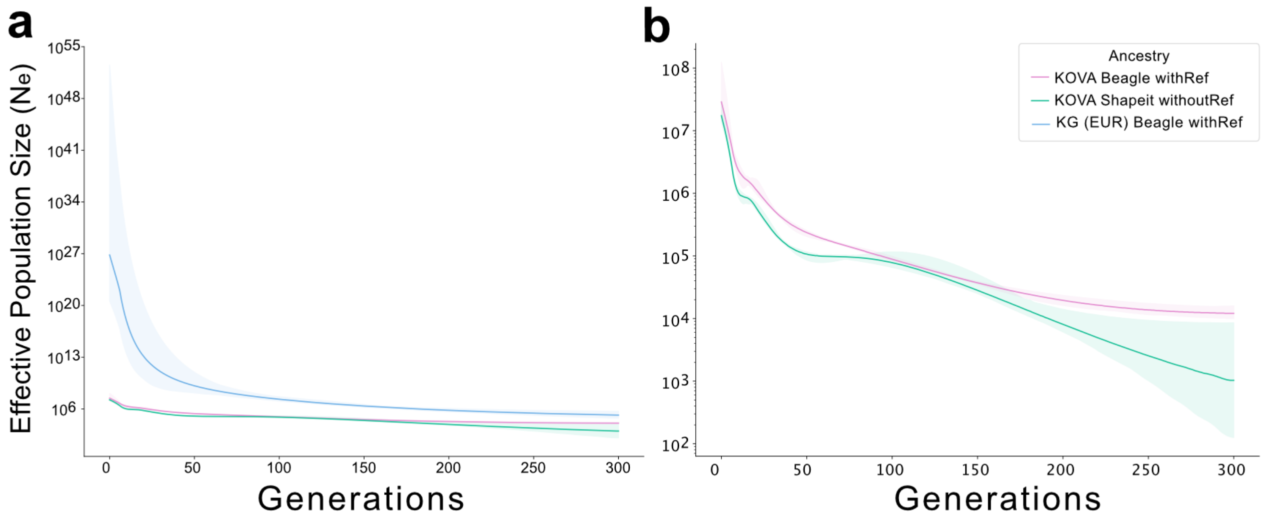

Supplementary Fig. 9. Estimated effective population size based on KOVA. (a) Effective Korean population size by generations before the present. (b) Estimated population size of Korean population calculated based on KOVA data, subset of (a).

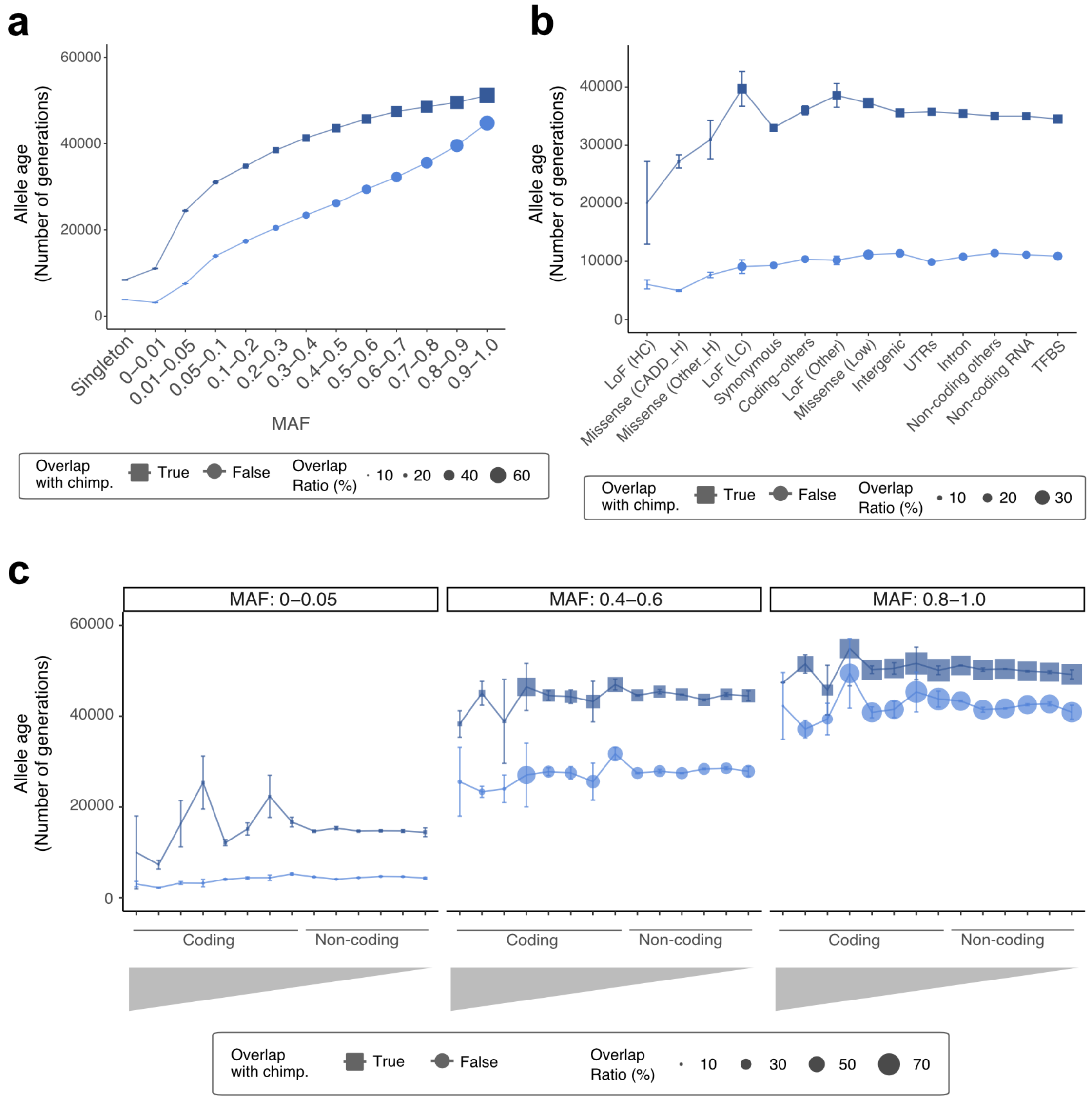

Supplementary Fig. 10. Allele ages of KOVA 2 variants based on KG data. (a) Allele ages by MAF, divided by the co-occurrence from chimpanzees (squares) or not (circles). (b) Allele ages by predicted function, divided by the co-occurrence from chimpanzees (squares) or not (circles). (c) Allele ages by MAF and predicted function. Three MAF intervals are displayed. The X-axis bins depicted in grey triangles in (c) are the same as that of (b)

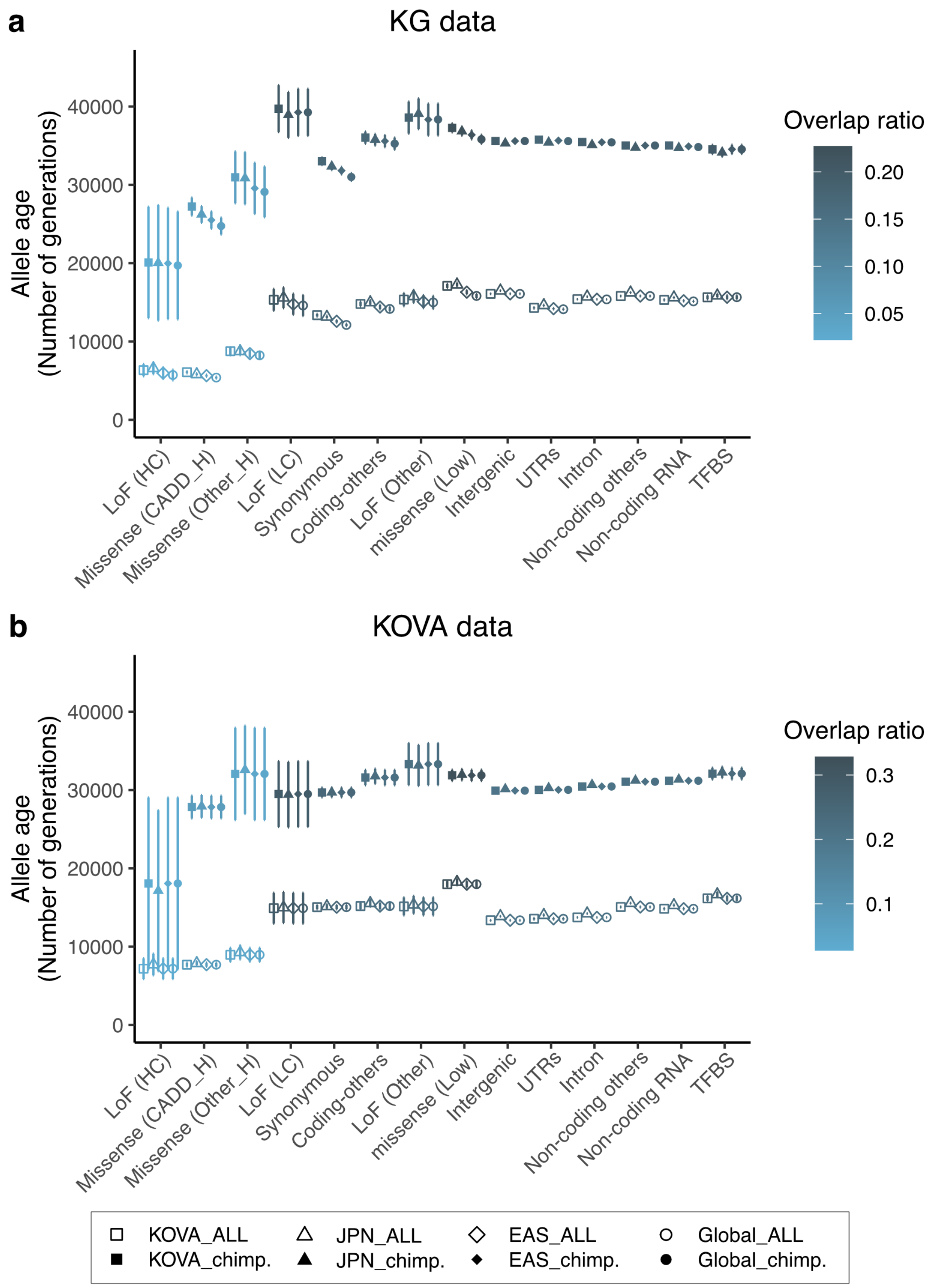

Supplementary Fig. 11. Allele ages of KOVA 2 variants by population. (a) Allele ages based on KG data by predicted function, divided by the co-occurrence from chimpanzees (filled) or not (blank). (b) Allele ages based on KOVA data by predicted function, divided by the co-occurrence from chimpanzees (filled) or not (blank).

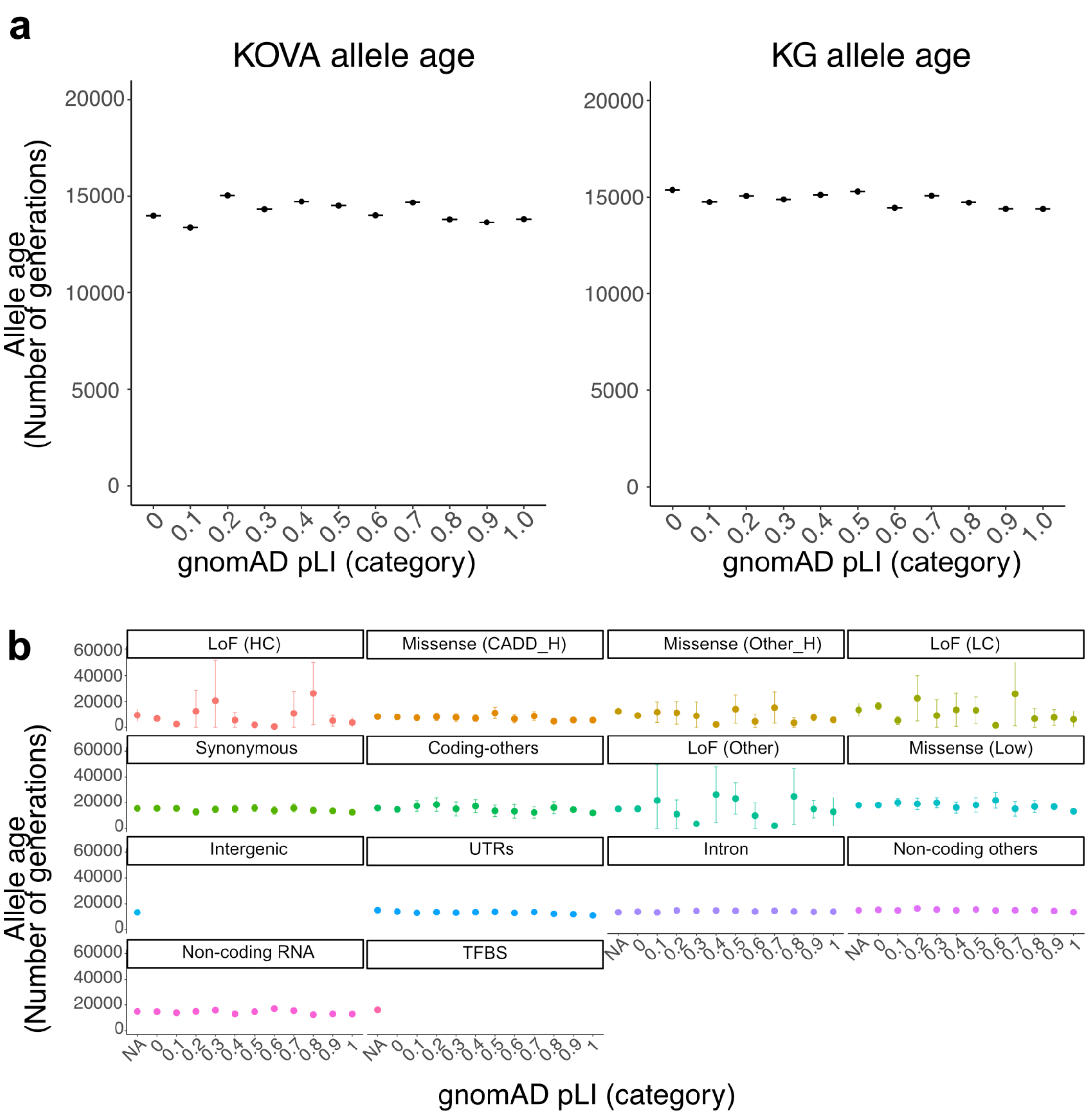

Supplementary Fig. 12. Allele age of KOVA 2 variants by pLI score. (a) Allele ages based on KOVA 2 data (left) and on KG data (right) by decile of gnomAD pLI score. NA represents variants without pLI scores. (b) Allele ages based on KOVA 2 data by pLI per predicted variant function.

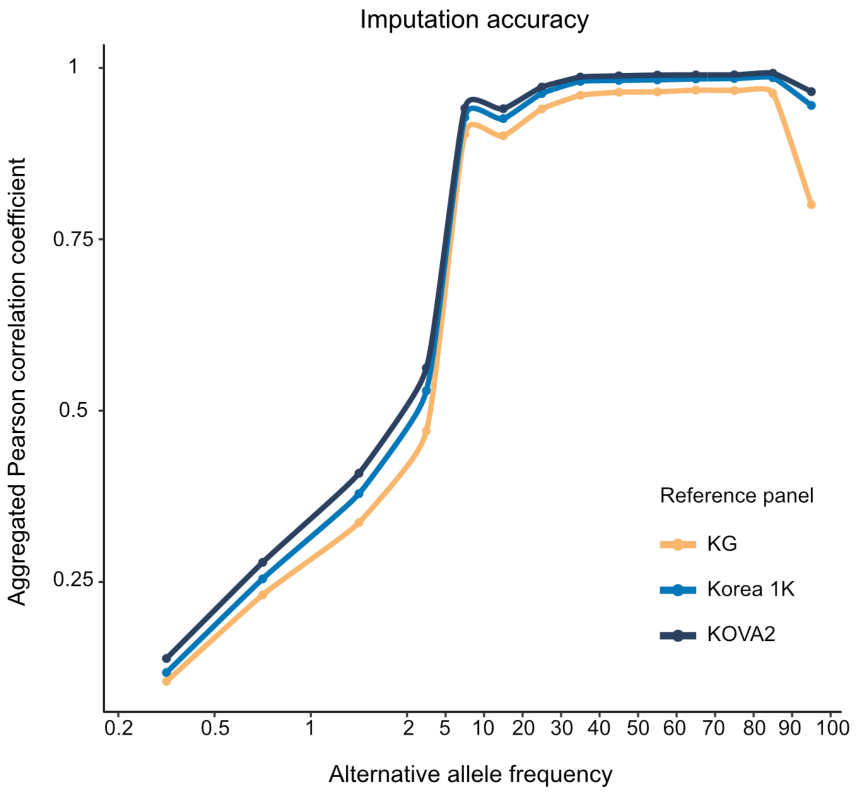

Supplementary Fig. 13. Imputation performance of KOVA 2 reference panel.

The aggregated Pearson correlation coefficient (R^2) between known genotypes from WGS data and imputed genotypes by the percentage of stratified alternative allele frequency.

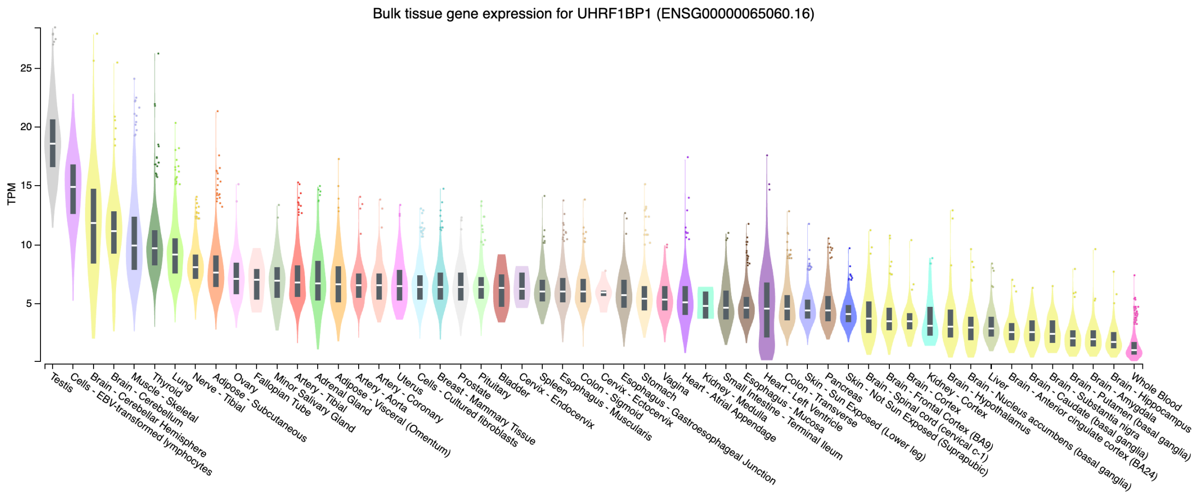

Supplementary Fig. 14. Tissue expression profile of *UHRF1BP1*. Displaying the highest expression in testes (on the far left; https://gtexportal.org/home/gene/UHRF1BP1).

Supplementary Table 1. Sample collection. Numbers denote number of samples after sample filtering. Note "KOVA I" denotes the data was also used in the first version of KOVA (Lee *et al., Sci Reports* 2017).

Group leader	Center	WES	WGS	Total	Note
Woong-Yang Park	Samsung Genome Institute	1,181	-	1,181	KOVA I
Jong Hwa Bhak	Ulsan National Institute of Science and Technology	-	903	903	-
Murim Choi	Seoul National University	587	23	610	-
Jong-Hee Chae	Seoul National University Children's Hospital	545	-	545	KOVA I
National Biobank of Korea	Korea Biobank Project	-	347	347	-
Young-Joon Kim	Yonsei University	-	324	324	-
The National Center for Medical Information and Knowledge	Clinical & Omics Data Archive (CODA)	-	299	299	-
Youngil Koh	Seoul National University Hospital	284	-	284	-
Daehyun Baek	Seoul National University	222	-	222	KOVA I
Sanghyuk Lee	Ewha Womans University	194	-	194	KOVA I
In-Jin Jang	Seoul National University	118	-	118	KOVA I
ETC		224	-	224	-
Heon Yung Gee	Yonsei University	45	-	45	-
Byung-Ok Choi	Samsung Medical Center	9	-	9	-
**Total**		**3,409**	**1,896**	**5,305**	**-**

Supplementary Table 2. Sample quality control process of WES data

Step	Condition	# of removed samples	Before	After
1	Ambiguous clinical status	306	4,235	3,929
2	Low coverage depth (meanCoverage < 40)	77	3,929	3,852
3	Ambiguous sex	92	3,852	3,760
4	Duplicated (kin > 0.35)	164	3,760	3,596
5	Related (0.1< kin <=0.35)	22	3,596	3,574
6	Ambiguous ethnicity	91	3,574	3,483
7	Het/hom ratio outlier (ratio > 1.8)	22	3,483	3,461
8	In both WES and WGS	52	3,461	3,409
	**Total**	**849**		**3,409**

Supplementary Table 3. Sample quality control process of WGS data

Step	Condition	Removed samples	Before	After
1	Low coverage depth (meanCoverage < 10)	165	2,396	2,231
2	Ambiguous sex	10	2,231	2,221
3	Duplicated (kin > 0.35)	144	2,221	2,077
4	Related (0.1< kin <=0.35)	149	2,077	1,928
5	Ambiguous ethnicity	32	1,928	1,896
	**Total**	**500**		**1,896**

Supplementary Table 4. Variant counts by functional class

Coding/ noncoding	Variant function	# of Singletons	# of non-singletons	total AC	# of known variants	# of novel variants
Coding	Transcript ablation	1	3	4	3	1
	Coding sequence variant	14	16	30	22	8
	Incomplete terminal codon variant	15	19	34	13	21
	Protein altering variant	130	31	161	43	118
	Stop retained variant	237	215	452	203	249
	Stop lost	616	567	1,183	502	681
	Inframe insertion	1,421	1,073	2,494	1,206	1,288
	Start lost	1,389	1,017	2,406	1,106	1,300
	Inframe deletion	4,043	3,021	7,064	3,780	3,284
	Splice acceptor variant	4,227	3,212	7,439	3,317	4,122
	Splice donor variant	5,243	4,618	9,861	4,887	4,974
	Stop gain	9,246	5,399	14,645	6,647	7,998
	Frameshift indel	12,239	6,583	18,822	6,157	12,665
	Splice region variant	43,437	43,699	87,136	49,383	37,753
	Synonymous variant	127,844	130,603	258,447	157,345	101,102
	Nonsynonymous SNV	274,693	226,818	501,511	270,422	231,089
Noncoding	Non coding transcript variant	-	1	1	1	-
	Regulatory region ablation	1	-	1	1	-
	TFBS ablation	112	121	233	143	90
	Mature miRNA variant	564	563	1,127	620	507
	TF binding site variant	41,715	48,332	90,047	58,693	31,354
	5 prime UTR variant	141,718	145,808	287,526	172,423	115,103
	3 prime UTR variant	341,553	361,410	702,963	436,082	266,881
	Regulatory region variant	529,226	619,532	1,148,758	737,657	411,101
	Non coding transcript exon variant	615,209	696,435	1,311,644	826,591	485,053
	Downstream gene variant	690,767	820,003	1,510,770	993,375	517,395

	Upstream gene variant	843,801	993,065	1,836,866	1,203,204	633,662
	Intergenic variant	4,929,501	5,832,094	10,761,595	6,406,273	4,355,322
	Intron	11,580,871	13,158,563	24,739,434	15,849,148	8,890,286
**Total**		**20,199,833**	**23,102,821**	**43,302,654**	**27,189,247**	**16,113,407**

Supplementary Table 5. Allele age by variant class

	All variants				Variants co-occurred from chimpanzee				Ratio (The co-occurrence from Chimp/All)			
**Variant class**	**kova allele age**	**kova allele cnt**	**kg allele age**	**kg allele cnt**	**kova allele age**	**kova allele cnt**	**kg allele age**	**kg allele cnt**	**kova allele age**	**kova allele cnt**	**kg allele age**	**kg allele cnt**
**LoF (HC)**	7,190	431	6,343	1,123	18,075	12	20,090	25	2.51	0.03	3.17	0.02
**Missense (Other_H)**	8,962	1,164	8,759	3,484	32,063	50	30,963	163	3.58	0.04	3.53	0.05
**Missense (CADD_H)**	7,704	12,567	6,075	36,661	27,832	945	27,227	1,815	3.61	0.08	4.48	0.05
**intergenic**	13,374	1,898,606	16,082	2,608,107	29,926	391,635	35,596	505,764	2.24	0.21	2.21	0.19
**intron**	13,742	4,353,161	15,409	6,593,118	30,459	915,522	35,470	1,233,526	2.22	0.21	2.30	0.19
**ncRNA**	14,846	190,890	15,313	326,043	31,192	40,277	35,030	56,884	2.10	0.21	2.29	0.17
**UTRs**	13,565	121,912	14,302	225,036	30,031	25,877	35,776	38,390	2.21	0.21	2.50	0.17
**nc-others**	15,093	747,565	15,805	1,234,003	31,091	163,582	35,018	229,091	2.06	0.22	2.22	0.19
**coding-others**	15,137	8,618	14,787	16,819	31,490	1,901	36,065	2,883	2.08	0.22	2.44	0.17
**synonymous**	15,062	18,811	13,362	48,377	29,723	4,600	32,997	8,260	1.97	0.24	2.47	0.17
**LoF (LC)**	14,914	400	15,325	840	29,496	114	39,728	171	1.98	0.29	2.59	0.20
**Missense (Low)**	17,979	10,403	17,105	24,103	31,886	3,367	37,284	5,475	1.77	0.32	2.18	0.23

Supplementary Table 6. The number of concordant or discordant variant pairs between PacBio and KOVA pipeline from a single sample.

	Genotype	KOVA2			Total
		**0/0**	**0/1**	**1/1**	
**PacBio**	0/0	46,220	3,820	610	293,949
	0/1	11,411	1,974,392	675	2,177,575
	1/1	742	3,613	1,548,512	1,734,669

Supplementary Table 7. The number of concordant or discordant variant pairs between NovaSeq and KOVA pipeline from a single sample.

	Genotype	KOVA2			Total
		0/0	0/1	1/1	
NovaSeq	0/0	-	-	-	-
	0/1	5,562	2,064,129	1,215	2,297,291
	1/1	56	1,028	1,495,016	1,629,156

Supplementary Table 8. Comparison of Sanger-validate calls and KOVA 2 calls.

Hom. Ref., homozygous reference; Het., heterozygous.

No.	KOVA2 sample ID	chr:position (hg38)	Sanger result	KOVA2 call			Con-cordant?
				Call	Ref. coverage	Nonref. coverage	
1	KVE0617	chr4:15059272	Hom. Ref.	Hom. Ref.	65	0	Yes
2	KVE0632	chr8:60743012	Hom. Ref.	Hom. Ref.	17	0	Yes
3	KVE0633	chr8:60743012	Hom. Ref.	Hom. Ref.	16	0	Yes
4	KVE0853	chr4:15059272	Hom. Ref.	Hom. Ref.	84	0	Yes
5	KVE0909	chr10:72551287	Hom. Ref.	Hom. Ref.	39	0	Yes
6	KVE2741	chr6:75087652	Hom. Ref.	Hom. Ref.	39	0	Yes
7	KVE2758	chr9:137162182	Hom. Ref.	Hom. Ref.	28	0	Yes
8	KVE2759	chr9:137162182	Hom. Ref.	Hom. Ref.	38	0	Yes
9	KVE2778	chr16:48361909	Hom. Ref.	Hom. Ref.	152	0	Yes
10	KVE2779	chr16:48361909	Hom. Ref.	Hom. Ref.	225	0	Yes
11	KVE2782	chr22:27751027	Hom. Ref.	Hom. Ref.	46	0	Yes
12	KVE2783	chr22:27751027	Hom. Ref.	Hom. Ref.	49	0	Yes
13	KVE2785	chr22:23787200	Hom. Ref.	Hom. Ref.	128	0	Yes
14	KVE2786	chr22:23787200	Hom. Ref.	Hom. Ref.	143	0	Yes
15	KVE2787	chr6:157184324	Hom. Ref.	Hom. Ref.	190	0	Yes
16	KVE2788	chr6:157184324	Hom. Ref.	Hom. Ref.	177	0	Yes
17	KVE2791	chr16:56354885	Hom. Ref.	Hom. Ref.	236	1	Yes
18	KVE2792	chr16:56354885	Hom. Ref.	Hom. Ref.	160	0	Yes
19	KVE2797	chr17:63964667	Hom. Ref.	Hom. Ref.	13	0	Yes
20	KVE2798	chr17:63964667	Hom. Ref.	Hom. Ref.	9	0	Yes
21	KVE2799	chrX:53382505	Hom. Ref.	Hom. Ref.	128	0	Yes
22	KVE2800	chrX:53382505	Hom. Ref.	Hom. Ref.	63	0	Yes
23	KVE2807	chr12:45837577	Hom. Ref.	Hom. Ref.	242	0	Yes
24	KVE2808	chr12:45837577	Hom. Ref.	Hom. Ref.	223	2	Yes
25	KVE2809	chr16:67539861	Hom. Ref.	Hom. Ref.	37	1	Yes
26	KVE2810	chr16:67539861	Hom. Ref.	Hom. Ref.	41	0	Yes
27	KVE2811	chr12:32733792	Hom. Ref.	Hom. Ref.	41	0	Yes
28	KVE2812	chr12:32733792	Hom. Ref.	Hom. Ref.	44	0	Yes

29	KVE2816	chrX:71564608	Hom. Ref.	Hom. Ref.	40	0	Yes
30	KVE2822	chr14:101980380	Hom. Ref.	Hom. Ref.	25	0	Yes
31	KVE2823	chr14:101980380	Hom. Ref.	Hom. Ref.	16	0	Yes
32	KVE2840	chr12:49033931	Hom. Ref.	Hom. Ref.	43	0	Yes
33	KVE2841	chr12:49033931	Hom. Ref.	Hom. Ref.	43	0	Yes
34	KVE3585	chr2:86252036	Hom. Ref.	Hom. Ref.	77	0	Yes
35	KVE3586	chr3:155084298	Hom. Ref.	Hom. Ref.	35	0	Yes
36	KVE3640	chr13:110176904	Hom. Ref.	Hom. Ref.	86	0	Yes
37	KVE3641	chr13:110176904	Hom. Ref.	Hom. Ref.	100	0	Yes
38	KVE3645	chr9:2081979	Hom. Ref.	Hom. Ref.	34	0	Yes
39	KVE3646	chr9:2081979	Hom. Ref.	Hom. Ref.	37	0	Yes
40	KVE3780	chr19:50323104	Hom. Ref.	Hom. Ref.	18	0	Yes
41	KVE3805	chr18:33740159	Hom. Ref.	Hom. Ref.	36	0	Yes
42	KVE3812	chrX:115165459	Hom. Ref.	Hom. Ref.	8	0	Yes
43	KVE3825	chr6:33451838	Hom. Ref.	Hom. Ref.	25	0	Yes
44	KVE3826	chr6:33451838	Hom. Ref.	Hom. Ref.	16	0	Yes
45	KVE3835	chrX:53234559	Hom. Ref.	Hom. Ref.	94	0	Yes
46	KVE3836	chrX:53234559	Hom. Ref.	Hom. Ref.	50	1	Yes
47	KVE3837	chr1:181651440	Hom. Ref.	Hom. Ref.	105	0	Yes
48	KVE3838	chr1:181651440	Hom. Ref.	Hom. Ref.	68	0	Yes
49	KVE4140	chr22:50675152	Hom. Ref.	Hom. Ref.	13	0	Yes
50	KVE4141	chr22:50675152	Hom. Ref.	Hom. Ref.	6	0	Yes
51	KVE4142	chr1:27552049	Hom. Ref.	Hom. Ref.	11	0	Yes
52	KVE4143	chr1:27552049	Hom. Ref.	Hom. Ref.	20	0	Yes
53	KVE4144	chr11:118758879	Hom. Ref.	Hom. Ref.	34	0	Yes
54	KVE4145	chr11:118758879	Hom. Ref.	Hom. Ref.	38	0	Yes
55	KVE0634	chr3:33018452	Het.	Het.	31	23	Yes
56	KVE0635	chr3:33068263	Het.	Het.	44	39	Yes
57	KVE0749	chr3:33018452	Het.	Het.	39	29	Yes
58	KVE0750	chr3:33018452	Het.	Het.	31	27	Yes
59	KVE0908	chr10:72551287	Het.	Het.	84	50	Yes
60	KVE2742	chr6:75087652	Het.	Het.	21	16	Yes
61	KVE2789	chr1:180274413	Het.	Het.	71	61	Yes
62	KVE2790	chr1:180274571	Het.	Het.	37	36	Yes

63	KVE2815 (F)	chrX:71564608	Het.	Het.	53	50	Yes
64	KVE2836	chr10:133364730	Het.	Het.	51	67	Yes
65	KVE2837	chr10:133373332	Het.	Het.	5	12	Yes
66	KVE3587	chr3:155084298	Het.	Het.	28	27	Yes
67	KVE3638	chr19:55137102	Het.	Het.	36	30	Yes
68	KVE3639	chr19:55134092	Het.	Het.	29	25	Yes
69	KVE3806	chr18:33740159	Het.	Het.	47	53	Yes
70	KVE3779	chr19:50323104	Het.	No call	1	8	No
71	KVE3811(F)	chrX:115165459	Het.	No call	1	4	No

Supplementary Table 9. The number of variants covered by WES, WGS only or by both methods.

MAF	Number of variants			Concordance
	Both	WES only	WGS only	
0.05-0.1	7,041	55	911	87.9%
0.1-0.2	8,200	60	1,090	87.7%
0.2-0.3	5,335	34	662	88.5%
0.3-0.4	4,147	30	557	87.6%
0.4-0.5	3,323	29	389	88.8%
0.5-0.6	2,949	23	329	89.3%
0.6-0.7	2,425	20	266	89.5%
0.7-0.8	2,063	8	233	89.5%
0.8-0.9	2,054	12	216	90.0%
0.9-1.0	2,657	24	271	90.0%
Total	40,194	295	4,924	88.5%
